# Parvalbumin Interneurons Are Differentially Connected to Principal Cells in Inhibitory Feedback Microcircuits along the Dorsoventral Axis of the Medial Entorhinal Cortex

**DOI:** 10.1523/ENEURO.0354-20.2020

**Published:** 2021-02-25

**Authors:** Sabine Grosser, Federico J. Barreda, Prateep Beed, Dietmar Schmitz, Sam A. Booker, Imre Vida

**Affiliations:** 1 Institute for Integrative Neuroanatomy, Charité – Universitätsmedizin Berlin, Berlin 10117, Germany; 2 Bernstein Center for Computational Neuroscience, Berlin 10115, Germany; 3 Neurowissenschaftliches Forschungszentrum, Charité-Universitätsmedizin, Berlin 10117, Germany; 4 Berlin Institute of Health, Berlin 10178, Germany; 5 NeuroCure Cluster of Excellence, Berlin 10117, Germany; 6 DZNE-German Center for Neurodegenerative Diseases, Berlin 10117, Germany; 7 Centre for Discovery Brain Sciences; 8 Simons Initiative for the Developing Brain; 9 Patrick Wild Centre for Autism Research, University of Edinburgh, Edinburgh EH8 9XD, United Kingdom

**Keywords:** entorhinal cortex, feedback inhibition, GABAergic interneurons, microcircuit, morphology, synapse

## Abstract

The medial entorhinal cortex (mEC) shows a high degree of spatial tuning, predominantly grid cell activity, which is reliant on robust, dynamic inhibition provided by local interneurons (INs). In fact, feedback inhibitory microcircuits involving fast-spiking parvalbumin (PV) basket cells (BCs) are believed to contribute dominantly to the emergence of grid cell firing in principal cells (PrCs). However, the strength of PV BC-mediated inhibition onto PrCs is not uniform in this region, but high in the dorsal and weak in the ventral mEC. This is in good correlation with divergent grid field sizes, but the underlying morphologic and physiological mechanisms remain unknown. In this study, we examined PV BCs in layer (L)2/3 of the mEC characterizing their intrinsic physiology, morphology and synaptic connectivity in the juvenile rat. We show that while intrinsic physiology and morphology are broadly similar over the dorsoventral axis, PV BCs form more connections onto local PrCs in the dorsal mEC, independent of target cell type. In turn, the major PrC subtypes, pyramidal cell (PC) and stellate cell (SC), form connections onto PV BCs with lower, but equal probability. These data thus identify inhibitory connectivity as source of the gradient of inhibition, plausibly explaining divergent grid field formation along this dorsoventral axis of the mEC.

## Significance Statement

Inhibition by parvalbumin basket cells (PV BCs) is essential for the emergence of grid firing in principal cells (PrCs) in the medial entorhinal cortex (mEC). The strength of PV BC-mediated inhibition decreases along the dorsoventral axis, in correlation with the grid field size of spatially tuned PrCs. In this study, to identify underlying cellular mechanisms, we combined electrophysiological recordings and neuroanatomical analysis investigating properties and connectivity of PV BCs in layer (L)2/3 of the rat mEC. While morphologic and physiological properties were largely uniform, interneuron (IN)-PrC connectivity was higher in the dorsal than ventral mEC. Thus, our results identify a difference in PV BC connectivity as source of the inhibitory gradient in the mEC with implications for grid size modulation.

## Introduction

The hippocampal formation, comprising the entorhinal cortex and hippocampus as its central structures, is a key component of the mammalian spatial navigation system (O’Keefe and Nadel, 1978). The medial entorhinal cortex (mEC) acts as the primary entry point of spatial information to the hippocampus, with layer (L)2/3 neurons projecting to the dentate gyrus, as well as to the CA1–CA3 areas (Varga et al., 2010; Witter et al., 2017). As such, spatially modulated neuronal activity has been described in essentially all areas of the formation, most notably as place cells in CA1 (O’Keefe, 1979), grid cells in L2/3 and L5 of the mEC (Sargolini et al., 2006; Boccara et al., 2010) and the dentate gyrus (Park et al., 2011). Grid cells are principal cells (PrCs) which display preferential action potential (AP) firing in hexagonally-arranged fields which overlay the two-dimensional environment (Hafting et al., 2005). Grid cells have been identified in the entorhinal cortex of all mammals so far investigated (Hafting et al., 2008; Yartsev et al., 2011). PrCs of mEC L2/3 comprise reelin-containing stellate cells (SCs), which are the canonical, highly spatially modulated grid cells, and calbindin-containing pyramidal cells (PCs) which also display spatial tuning (Sargolini et al., 2006; Tang et al., 2014; Tennant et al., 2018). The spatial tuning of PrCs is maintained along the dorsoventral extent of the mEC (Ray et al., 2014); however, grid fields are not uniform, but show a gradient in the scale and size of grid fields. In the dorsal mEC, grid fields are small with higher spatial resolution, whereas in the ventral mEC, grid fields are larger, perhaps corresponding to different roles in spatial navigation (Hafting et al., 2005; Brun et al., 2008).

Despite these known features of mEC neuronal activity, the cellular and network mechanisms leading to physiological divergence of grid field size along the dorsoventral axis are not fully understood. Intrinsic physiology of L2/3 PrCs displays dorsoventral asymmetry (Heys et al., 2010) tuning dorsal PrCs to higher theta frequencies (Pastoll et al., 2012), whereas γ frequency activity is of higher power in the dorsal mEC (Beed et al., 2013). Although L2/3 PrCs themselves are interconnected (Winterer et al., 2017), they form a sparse network which is overlain by a rich population of local GABAergic inhibitory interneurons (INs; Couey et al., 2013; Buetfering et al., 2014; Berggaard et al., 2018). These local feedback inhibitory microcircuits, in particular fast-spiking parvalbumin basket cells (PV BCs), have been proposed as a key component of grid cell organization, as both stable grid firing and spatial organization are dependent on IN activity (Pastoll et al., 2013; Miao et al., 2017). PV BCs produce fast-spiking trains of APs and mediate both feed-forward and feedback inhibition onto the perisomatic compartments of PrCs (Jones and Bühl, 1993; Berggaard et al., 2018). Their postsynaptic effects control the precise timing of APs in PrCs and contribute to the generation of coherent network oscillations (Pouille and Scanziani, 2001; Bartos et al., 2007). However, despite their well-established involvement in perisomatic inhibition in the mEC, little is known regarding their electrophysiological and neuroanatomical properties.

GABAergic inhibition in L2 of the mEC shows distinct properties along the dorsoventral axis, in good correlation with the gradient of grid activity. Although the density of PV BCs was comparable in the two subregions, they produce stronger postsynapticeffects and target PrCs over a wider area in the dorsal mEC compared with those in ventral domains (Beed et al., 2013), which may explain the difference in inhibitory strength along the dorsoventral axis (Berggaard et al., 2018). Therefore, in this study we examined the morphology, intrinsic physiology and synaptic connectivity of PV BCs in the dorsal and the ventral mEC in a comparative manner, by performing whole-cell patch clamp recordings from single PV BCs as well as synaptically-coupled pairs of INs and PrCs in acute rat brain slices, combined with *post hoc* visualization and morphologic analysis.

## Materials and Methods

All experiments were performed in accordance with the [European and national guidelines (German Animal Welfare Act)] and institutional guidelines in the presence of permissions from local authorities [T-0215/11, LaGeSo Berlin, Germany]. Recordings were performed on acute brain slices from 18- to 27-d-old male and female Wistar rats, expressing the yellow-shifted Venus fluorescent protein under the VGAT promoter (Uematsu et al., 2008), housed on a 12/12 h light/dark cycle with *ad libitum* food and water.

### Electrophysiological recordings from acute brain slices

**Figure 1.**
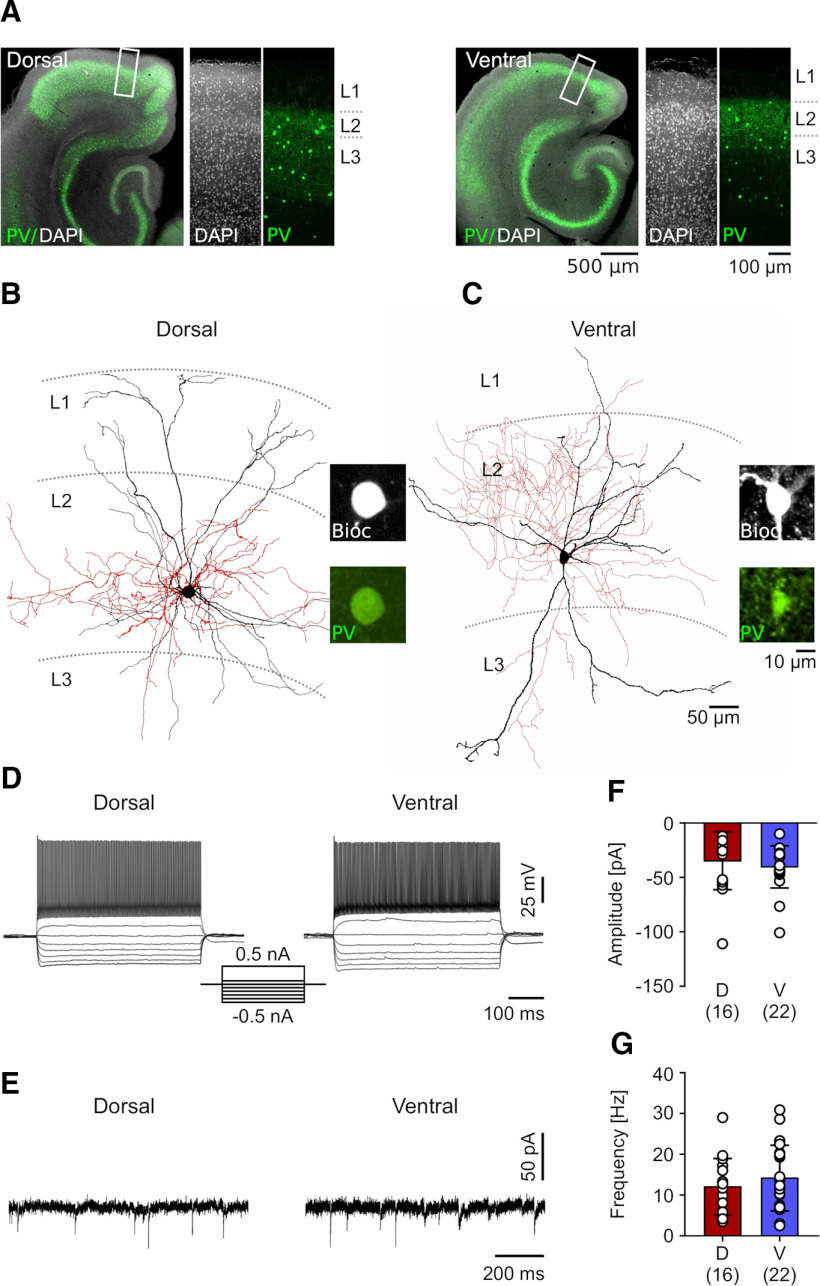
Morphology and physiological signature of fast-spiking PV BCs in the dorsal and ventral mEC. ***A***, Representative confocal images from the slices of the dorsal (left panel) and ventral mEC (right panel) immunostained for PV (in green pseudocolor) and counterstained by DAPI (grayscale). Higher-magnification images illustrate the superficial layers (L1–L3) of the mEC corresponding to the white rectangles in the overview images in the separate channels. ***B***, ***C***, Reconstructions of biocytin-filled fast-spiking PV BCs recorded in L2/3 of the dorsal (***B***) and ventral mEC (***C***). Soma and dendrites of the INs are depicted in black, axons in red; boundaries of the layers (L1–L3) are indicated by dotted lines. Insets on the right illustrate the PV immunoreactivity (in green) in the biocytin-filled somata of the INs (Bioc, grayscale). ***D***, ***E***, Voltage responses of the two visualized PV BCs to hyperpolarizing (−500 to −100 pA) and depolarizing current pulses (100 and 500 pA, 500 ms in duration, see inset in the middle). Note the fast-spiking non-accommodating AP discharge pattern in response to the strong depolarizing current pulse in both INs. ***F***, ***G***, Summary bar charts of the amplitude (***F***) and frequency of spontaneous EPSPs in dorsal (D, red bars) and ventral PV BCs (V, blue bars). Data from individual neurons are superimposed as open circles; numbers of recorded neurons are indicated in parenthesis under the bars.

Acute brain slices were produced as described earlier (Booker et al., 2014). Briefly, rats were anesthetized (3% Isoflurane, Abbott) and then decapitated. Brains were quickly removed and transferred to carbogenated (95% O_2_/5% CO_2_) ice-cold sucrose-ACSF containing the following: 87 mm NaCl, 2.5 mm KCl, 25 mm NaHCO_3_, 1.25 mm NaH_2_PO_4_, 25 mm glucose, 75 mm sucrose, 7 mm MgCl_2_, 0.5 mm CaCl_2_, 1 mm Na-pyruvate, and 1 mm ascorbic acid. Horizontal brain slices (300 μm thick) were cut using a vibratome (VT1200 S, Leica). Dorsal and ventral mEC slices, corresponding to ∼4.6–5.6 and 6.8–7.8 mm from the dorsal surface of the brain, respectively (Paxinos, 1998), were separately collected (Fig. 1*A*), placed in a submerged holding chamber filled with carbogenated sucrose ACSF at 32–34°C for 30 min and then at room temperature until recording. Experiments were alternated between dorsal and ventral slices to prevent bias because of slice condition.

For recording, slices were transferred to a submerged chamber and superfused with prewarmed, carbogenated ACSF containing the following: 125 mm NaCl, 2.5 mm KCl, 25 mm NaHCO_3_, 1.25 mm NaH_2_PO_4_, 25 mm glucose, 1 mm MgCl_2_, 2 mm CaCl_2_, 1 mm Na-pyruvate, and 1 mm ascorbic acid. The bath temperature was set to 32–34°C with a perfusion rate of 12–13 ml/min. Slices were visualized using an upright microscope (BX-51WI; Olympus) equipped with infrared differential inference contrast optics and a digital camera (Zyla CMOS, Andor). PV BCs were identified as Venus-positive large multipolar cells visualized by epifluorescent illumination delivered via a fixed wavelength LED source (λ = 514 nm, OptoLED, Cairn Research). L2/3 PrCs were preselected for recording within the cortical layer as neurons lacking Venus fluorescence. The boundary of mEC was identified based on previous anatomic studies (Canto and Witter, 2012).

Whole-cell patch-clamp electrodes were produced from borosilicate glass capillaries (outer diameter, 2 mm; inner diameter, 1 mm; Hilgenberg) using a horizontal puller (P-97, Sutter Instruments) and filled with an intracellular solution consisting of the following: 130 mm K-gluconate, 10 mm KCl, 10 mm HEPES, 10 mm EGTA, 2 mm MgCl_2_, 2 mm Na_2_ATP, 0.3 mm Na_2_GTP, 1 mm Na_2_creatine, and 0.1% mm biocytin (adjusted to pH 7.3 and 315 mOsm), giving a series resistance of 2.5–4 MΩ. All recordings were performed with an Axopatch 700B amplifier (Molecular Devices), filtered online at 10 kHz with the built-in two-pole Bessel filter, and digitized at 20 kHz (National Instruments). Following breakthrough into whole-cell configuration, we characterized resting membrane potential and intrinsic physiological properties in current-clamp mode. The apparent membrane time constant and input resistance were measured in averaged responses (30 traces) to a small hyperpolarizing current pulse (10 pA, 500 ms in duration). AP properties were determined from small depolarizing current steps to 10 pA above rheobase (10 pA steps, 500 ms in duration), AP and afterpotential amplitudes were measured from threshold. Discharge frequency and sag response were characterized using hyper- to depolarizing current pulses (a family of −500 to 500 pA in 100 pA steps, followed by a single step to 1 nA, 500 ms in duration for FS BCs, and a family of −250 to 250 pA in 50 pA steps, 500 ms in duration for PrCs). Maximal discharge frequency of FS BCs was measured in responses to 1 nA pulses. The voltage “sag” amplitude of PrCs was measured in averaged responses to −250 pA pulses. We calculated the interspike interval (ISI), as the ratio between the length of the first ISI and the second ISI in a pulse train elicited by a 250-pA current pulse. The latency to the fist spike was taken as the time between the start of the current pulse and the threshold crossing to the first AP. For voltage clamp recordings, neurons were kept at a holding potential of −65 mV. Specific membrane capacitance was measured with hyperpolarizing voltage pulses (−10 mV, 500 ms in duration). Finally, the spontaneous synaptic input onto PV BCs was characterized by recording EPSCs for 1 min. Cells were excluded if resting membrane potential was more depolarized than −45 mV, initial series resistance >30 MΩ or >20% change occurred in series resistance over the course of the recording. The liquid junction potential was not corrected for.

Paired recordings between PV BCs and L2/3 PrCs were performed to determine connectivity and synaptic properties, as described earlier (Booker at al., 2014). Briefly, a fast-spiking putative PV BC was recorded in current-clamp mode and a postsynaptic Venus-negative cell was patched in close proximity (<200 μm distal), and recorded in voltage clamp at a holding potential of −60 mV to record outward GABA_A_ receptor-mediated unitary IPSCs (estimated reversal potential for Cl^–^ = −70 mV). The series resistance was compensated to 80%. APs were produced in the IN by short depolarizing pulses (2 nA amplitude, 2 ms in duration) and the postsynaptic cell recorded simultaneously. The reciprocal connectivity was then tested from the PrC to the IN under the same conditions, albeit with the PrCs in current-clamp and the IN in voltage clamp mode. Depending on the stability of the IN recording, multiple postsynaptic PrCs (2 to 4) were recorded for each IN. Synaptic connectivity was characterized in response to 50 APs evoked in the presynaptic neuron, with failures of transmission determined as IPSC amplitudes less than twice the standard deviation of baseline fluctuations measured within a 20 ms window before the AP. IPSCs were detected and their amplitudes determined in a 10 ms window directly following the AP.

All electrophysiological data were acquired online using the open-source WinWCP software package (courtesy of J. Dempster, Strathclyde University, Glasgow, UK; http://spider.science.strath.ac.uk/sipbs/software_ses.htm) and off-line analysis was performed using the Stimfit software (courtesy of C. Schmidt-Hieber; http://stimfit.org; Schlögl et al., 2013).

PrCs were classified as PCs or SCs by principal component analysis following the approach by (Fuchs et al., 2016), using three physiological parameters: depolarizing afterpotential (dAP), latency and ISI, calculated previously (Table 3, double asterisk), as well as the presence or absence of an apical dendrite. For the clustering, we standardized the parameters and ran the principal component function “princomp” in R (https://rstudio.com) using a custom script. We grouped the resulting data on the first two principal components, which were responsible for >90% of the sample variance as determined by the elbow method and clustered it using the “ward” hierarchical clustering approach using custom written scripts in Python 3.7.

### Neuronal visualization and immunohistochemistry

Following recording, cells were resealed by obtaining outside-out patches and slices immersion-fixed in 4% paraformaldehyde (PFA) in 0.1 m phosphate buffer (PB; pH 7.4) at 4°C for 24–48 h. Slices were then transferred to fresh PB and stored for a maximum of three weeks. Before immunohistochemical processing, slices were rinsed in PB, followed by PBS (0.9% NaCl) and blocked in PBS containing 10% normal goat serum (NGS), 0.3% Triton X-100 and 0.05% NaN_3_ for 1 h. Slices were then incubated with a monoclonal mouse antibody raised against PV (1:5000, PV-235, Swant) in PBS containing 5% NGS, 0.3% Triton X-100 and 0.05% NaN_3_ for 72 h at 4°C. Slices were then rinsed in PBS and a fluorescent-conjugated secondary antibody was applied (goat anti-mouse IgG, Alexa Fluor-405, 1:1000, Invitrogen) in combination with fluorescent-conjugated streptavidin (Alexa Fluor-647, 1:1000, Invitrogen), in a PBS solution containing 3% NGS, 0.1% Triton X-100 and 0.05% NaN_3_ for 24 h at 4°C. Slices were rinsed in PBS and then desalted in PB before being mounted in on glass slides (Fluoromount-G, Southern Biotech) with a 300-μm-thick agar spacer, cover-slipped, sealed and stored at 4°C before imaging.

### Confocal imaging and reconstruction

Recorded cells and pairs of cells were imaged on a laser scanning confocal microscope (FV1000, Olympus). First, a low magnification (4× air immersion, Olympus) overview image was taken to confirm the cellular localization to the mEC, then high resolution z-stacks were made with a 30× silicone immersion lens (N.A. 1.05, UPlanSApo, Olympus) over the whole extent of the cell (1 μm axial steps). To confirm PV neurochemical identity of the recorded cells, a high-magnification image (60× objective, N.A. 1.2, Olympus) was taken over the soma, proximal dendrites and axon collaterals. Cells were deemed to be PV BCs if the soma, proximal dendrites, or axon terminals showed labeling for PV and presented axonal baskets characteristic of BC morphology (Fig. 1*B*,*C*). Axo-axonic neurons with typical vertical axon cartridges (Somogyi et al., 1982; Defelipe et al., 1985) were excluded from the analysis.

Images were analyzed offline using the FIJI software package (http://fiji.sc/wiki/index.php/FIJI). Image stacks were stitched, the cells reconstructed and volume filled using the Simple Neurite Tracer plug-in (Longair et al., 2011). Image scaling and measurement of morphologic parameters were performed using a custom script within the NEURON environment (Hines and Carnevale, 1997). Comparative morphology was assessed by overlaying dendritic and axonal arborizations from reconstructed cells from either dorsal or ventral mEC in MATLAB (2013b, MathWorks). Superimposed images were smoothed using a Gaussian filter, based on a standard deviation of seven and presented on a logarithmic scale. The 2D correlation indices were calculated using the ‘corr2’ MATLAB function. Axonal bouton density was determined in a subset of cells, randomized and presented for blind counting. The number of boutons was counted on 8–10 straight, 25 to 70 μm-long axon segments from each cell and the corresponding bouton density along the axon was calculated. The total number of boutons was estimated from the length of the axon and the bouton density calculated for the respective cells.

### Statistics and analysis

Data are shown as the mean ± SEM unless indicated otherwise. Data were tested for normality (Shapiro–Wilk test) and, where normally distributed, assessed with Student’s *t* test for statistical significance. Non-normal data were tested using the Mann–Whitney *U* non-parametric test. Fisher’s exact test was used for analysis of connectivity to compare contingencies and two-way ANOVA for testing significance between three and more groups. For the reciprocal connectivity ratios were obtained from the variance calculated from the first order Taylor expansion of the samples (Stuart and Ord, 2010). For all tests significant differences were assumed if *p *<* *0.05 and significance level was set to *p <* *0.05, **p <* *0.01, and ***p <* *0.001.

## Results

To investigate the anatomic and physiological diversity among PV BCs at the cellular level, which could underlie differences in inhibitory output along the dorsoventral axis of the mEC (Beed et al., 2013), we performed whole-cell patch-clamp recordings from the dorsal and the ventral mEC in acute brain slices (Fig. 1*A*). PV BCs were selected in L2/3 based on their expression of Venus-YFP and multipolar appearance of their cell bodies, when observed under the epifluorescent microscope. During the recordings, we identified 98 fast-spiking INs (45 cells in the dorsal and 53 cells in the ventral mEC from 40 rats), out of which 89 were subsequently confirmed to be immunoreactive for PV with morphologic features of BCs (Fig. 1*B*,*C*; see further detail below). Nine putative axo-axonic cells, displaying vertical axon cartridges, were excluded from this study.

### Similar intrinsic physiological properties and excitability of L2/3 PV BCs in dorsal and ventral mEC

A possible explanation for the differences in lateral inhibition between dorsal and ventral mEC (Beed et al., 2013) may arise from INs possessing divergent intrinsic responses to depolarizing currents. To investigate this possibility, we characterized both the passive and active properties of the recorded INs. PV BCs showed no difference in any of their passive properties between dorsal and ventral mEC: their resting membrane potential, membrane resistance, time constant and cell capacitance (Table 1). In response to suprathreshold depolarizing current pulses, PV BCs consistently fired non-accommodating trains of APs at high frequency in both dorsal and ventral mEC; Fig. 1*D*). There was no apparent difference in the excitability of PV BCs between the two subregions in terms of the voltage threshold, rheobase or their maximum firing frequency at 1 nA either (Table 1). Finally, given that the excitability of a given neuron is directly related to its synaptic input, we asked whether the spontaneous EPSCs arriving onto PV BCs in the dorsal mEC were stronger than those arriving in the ventral mEC (Fig. 1*E*). We observed no difference in either amplitude or frequency of spontaneous EPSCs between the two IN groups (Fig. 1*E–G*). Together, these data show that PV BCs display comparable intrinsic excitability and spontaneous excitatory synaptic inputs, independent of their location along the dorsoventral axis of the mEC.

**Table 1:** Passive and active physiological properties of dorsal and ventral PV BCs

	Dorsal	Ventral	*p* value
Passive properties			
Resting membrane potential (mV)	−66.0 ± 1.7 (25)	−67.7 ± 1.6 (32)	0.46
Input resistance (MΩ)	83.1 ± 8.0 (25)	95.3 ± 8.0 (28)	0.29
Membrane time constant (ms)	11.5 ± 1.5 (17)	14.9 ± 2.7 (23)	0.87
Membrane capacitance (pF)	16.7 ± 2.9 (13)	13.1 ± 1.4 (16)	0.28
Active properties			
Rheobase (pA)	205.3 ± 48.2 (11)	164.7 ± 34.9 (15)	0.50
Voltage threshold (mV)	−38.2 ± 1.0 (11)	−40.8 ± 1.2 (15)	0.10
AP amplitude (mV)^*^	53.5 ± 3.6 (11)	64.4 ± 4.0 (15)	0.07
AP rise time (ms)	0.99 ± 0.55 (11)	0.33 ± 0.19 (14)	0.57
AP maximum rise rate (mV/ms)	308.2 ± 27.2 (11)	326.1 ± 16.1 (15)	0.56
AP maximum decay rate (mV/ms)	185.1 ± 20.0 (11)	161.1 ± 14.3 (15)	0.96
Half-height width (ms)	0.44 ± 0.04 (11)	0.49 ± 0.03 (15)	0.22
Fast AHP amplitude (mV)^*^	−20.4 ± 0.94 (12)	−21.1 ± 0.79 (15)	0.58
Discharge frequency at 1 nA** **(Hz)	298.0 ± 30.9 (16)	252.1 ± 17.5 (22)	0.21
Spontaneous synaptic input			
Spontaneous EPSC frequency (Hz)	12.2 ± 6.8 (25)	14.4 ± 7.6 (32)	0.38
Spontaneous EPSC amplitude (pA)	−35.5 ± 25.7 (25)	−38.3 ± 23.4 (32)	0.73

Results shown as mean ± SEM; number of analyzed neurons are shown in parenthesis.

* Measured from threshold.

### Dorsal and ventral PV BCs show similar dendritic and axonal morphologies

Another plausible explanation for dorsoventral differences in lateral inhibition might be a divergence in PV BC morphology; allowing individual neurons to receive and transmit information to narrower *versus* wider fields of the mEC. Therefore, we next analyzed the morphology of the recorded and visualized neurons (Fig. 1*B*,*C*). PV BCs from both dorsal and ventral mEC had 3 to 9 aspiny or sparsely spiny primary dendrites emerging from the soma, spanning L3–L1, often with a dendritic tuft reaching the pial surface. The axon emerged typically from the soma and formed a dense local arbor in L2/3 around the cell bodies of neighboring neurons, consistent with the INs being BCs (Fig. 1*B*,*C*). While we observed differences in their morphology, including the extent of the axon, this variability was similarly present in both dorsal and ventral mEC.

**Figure 2.**
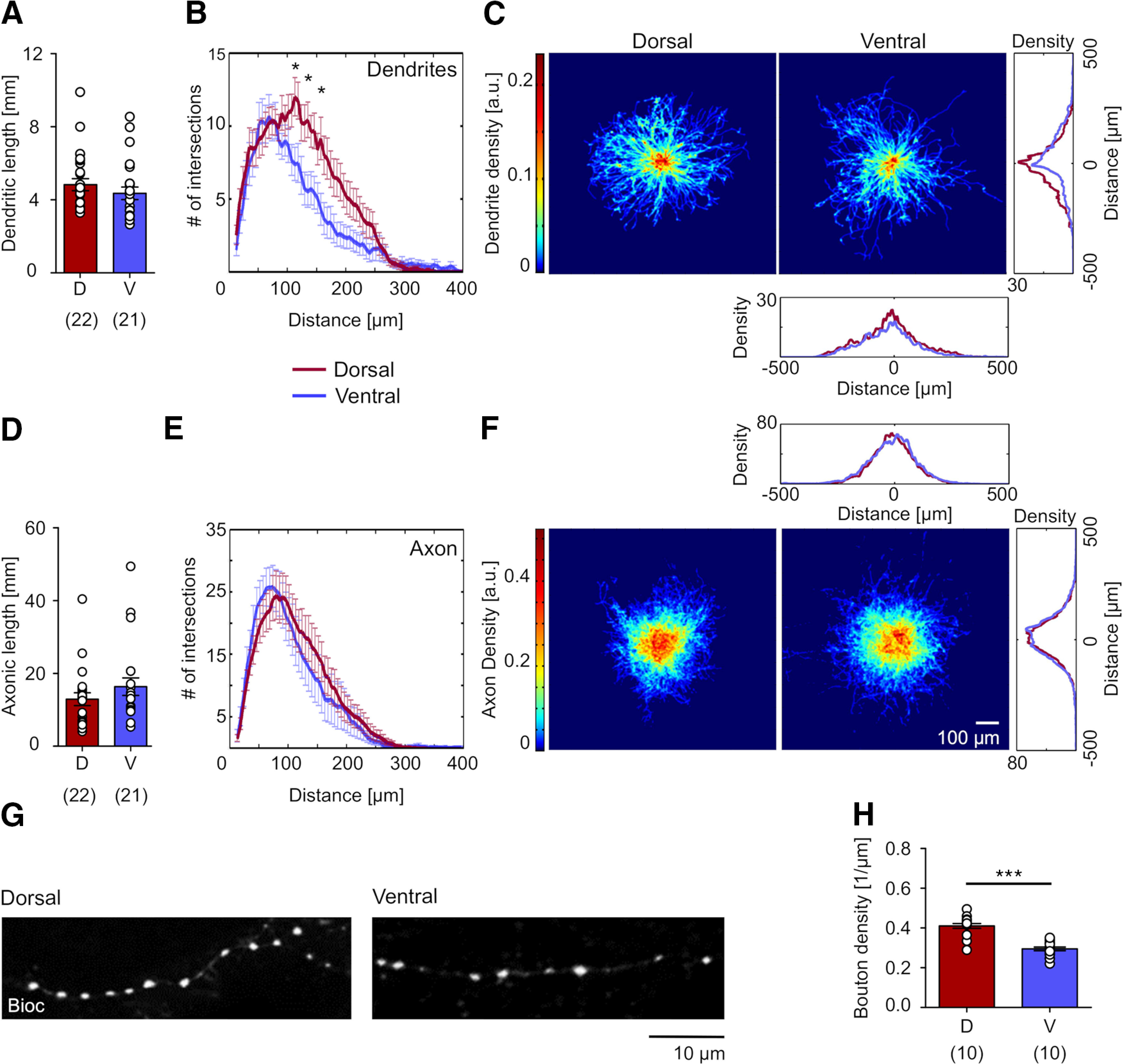
Neuroanatomical properties of PV BCs in the dorsal and ventral mEC. ***A***, ***D***, Summary bar charts of the length of dendrites (***A***) and axons (***D***) of dorsal (D, red bars) and ventral PV BCs (V, blue bars). Data from individual neurons is superimposed as open circles; numbers of analyzed neurons are indicated in parenthesis under the bars. ***B***, ***E***, Sholl analysis of the dendritic (***B***) and axonal arbors (***E***) of dorsal (in red) and ventral PV BCs (in blue). Sholl radius was set to 25 μm, and significance was tested using Fischer’s exact test; asterisks indicate significant differences at the level of *p* = 0.05. ***C***, ***F***, Cumulative heat maps of the spatial densities of dendritic (***C***) and axonal distributions (***F***) for dorsal (left) and ventral PV BCs (right). Individual INs viewed in the plane of the slices were aligned with their somata to the middle of the plots. The color code for the density (in arbitrary units) is on the left. One-dimensional density plots on the right and bottom illustrate the spatial integrals of the densities along the *x*- and *y*-axes, respectively, for dorsal (in red) and ventral INs (in blue). ***G***, A confocal image of axon collaterals of intracellularly-filled PV BCs displaying varicosities in the dorsal (left) and ventral mEC (right). ***H***, Summary bar chart of the density of varicosities along axon collaterals of PV BCs from the dorsal (D, red bars) and ventral mEC (V, blue bars). Data from individual neurons are superimposed as open circles; numbers of analyzed neurons are indicated in parenthesis under the bars. Statistical significance: *p < 0.05 and ***p < 0.001.

To determine whether the distributions of dendritic or axonal arbors were different between dorsal and ventral mEC, we reconstructed a subset of the INs and performed morphometric analysis (Fig. 2, Table 2). PV BCs from the dorsal mEC had a total dendritic length of 4.8 ± 0.3 mm (22 PV BCs from 18 rats), comparable to those in the ventral mEC with 4.4 ± 0.3 mm (21 PV BCs from 17 rats; *p* = 0.29, Mann–Whitney *U* test; Fig. 2*A*). Sholl analysis revealed a higher number of segments in proximal dendrites of dorsal PV BCs at 100–200 μm of the soma compared with ventral INs (*F* = 22.4, two-way ANOVA; Fig. 2*B*). To examine the extent, symmetry, and distribution of dendrites, we next analyzed the dendritic volume density (Fig. 2*C*). Superimposing PV BC dendritic arbors with the somata as reference point, we calculated the mean volume density for the reconstructed neurons. This analysis revealed no discernible differences in the distribution pattern between the two groups for the transverse and vertical axes (Fig. 2*C*, insets), confirming that dendrites in dorsal and ventral PV BCs had very similar density distributions. A quantitative comparison of the volume density maps revealed that dendrites of reconstructed neurons in dorsal and ventral regions are highly similar (*r* = 0.75, 2D correlation coefficient).

**Table 2:** Anatomical properties of dorsal and ventral PV BCs

	Dorsal	Ventral	*p* value
Dendrites			
Total dendritic length (μm)	4828.4 ± 328.6 (22)	4357.8 ± 343.2 (21)	0.29
Lateral extent (μm)	418.1 ± 25.2 (21)	429.5 ± 27.5 (19)	0.59
Vertical extent (μm)	425.9 ± 22.6 (21)	402.5 ± 30.8 (19)	0.46
Dendritic field (μm^2^)	184 609 ± 15 815 (21)	181 726 ± 22 509 (19)	0.92
Axon			
Total axonal length (μm)	12,919 ± 1737 (21)	16,332 ± 2418 (19)	0.35
Lateral extent (μm)	411.5 ± 31.0 (21)	430.5 ± 30.9 (19)	0.67
Vertical extent (μm)	377.4 ± 27.3 (21)	421.3 ± 37.0 (19)	0.34
Axonal field (μm^2^)	158,754 ± 14,854 (21)	189,212 ± 22,513 (19)	0.43
Varicosity density (1/μm)	0.41 ± 0.01 (10)	0.30 ± 0.01 (10)	<0.0001

Results shown as mean ± SEM; the numbers of analyzed neurons are shown in parenthesis.

We next performed the same analysis of the axonal distribution of PV BCs along the dorsoventral axis of the mEC. The mean axon length in dorsal PV BCs was 12.9 ± 1.7 mm (22 INs from 18 rats) and of ventral cells was 16.3 ± 2.4 mm (19 INs from 14 rats), which was not statistically different (*p* = 0.35, Mann–Whitney *U* test; Fig. 2*D*). Sholl analysis (Fig. 2*E*) and volume density plots of the axonal distributions (Fig. 2*F*) showed very similar spatial structure and distribution of the axons. This was further reflected by a high correlation between the volume density maps for dorsal and ventral INs (*r* = 0.91, 2D correlation coefficient) as well as strongly overlapping transverse and vertical projections of the density distributions (Fig. 2*F*, insets).

As the length and distribution of the axon were not different between the two subregions, we next examined the density of boutons along the axon of PV BCs, as an indicator for the number of synapses formed. In the dorsal mEC, we found a high density of putative inhibitory boutons on PV BC axons (mean density: 0.41 ± 0.01 μm^−1^, 87 axon segments from 10 cells from nine rats), 41% higher than that in the ventral mEC (mean density: 0.30 ± 0.01 μm^−1^, 85 axon segments from 10 PV BCs from 10 rats; *p* < 0.0001, Student’s *t* test on cell averages; Fig. 2*G*,*H*). As such, a higher number of synapses may be formed by the axon of dorsal PV BCs. Indeed, estimates using the obtained density values of putative synaptic boutons and the corresponding axonal lengths of the INs indicated that the number of potential synaptic contacts made by dorsal INs was 7512 ± 51, in ventral PV BCs this estimate was lower at 5127 ± 39 (*p* = 0.023, Student’s *t* test).

Taken together, these data demonstrate that the dendritic distribution of PV BCs show minor region-specific differences along the dorsoventral axis of the mEC (Table 2). Despite the axon of PV BCs displaying a comparable length and broadly similar lateral distribution in both mEC subfileds, the number of putative synapses formed by this axon is greater in the dorsal mEC.

### PV BCs along the dorsoventral axis of the mEC are differentially connected to PrCs

**Figure 3.**
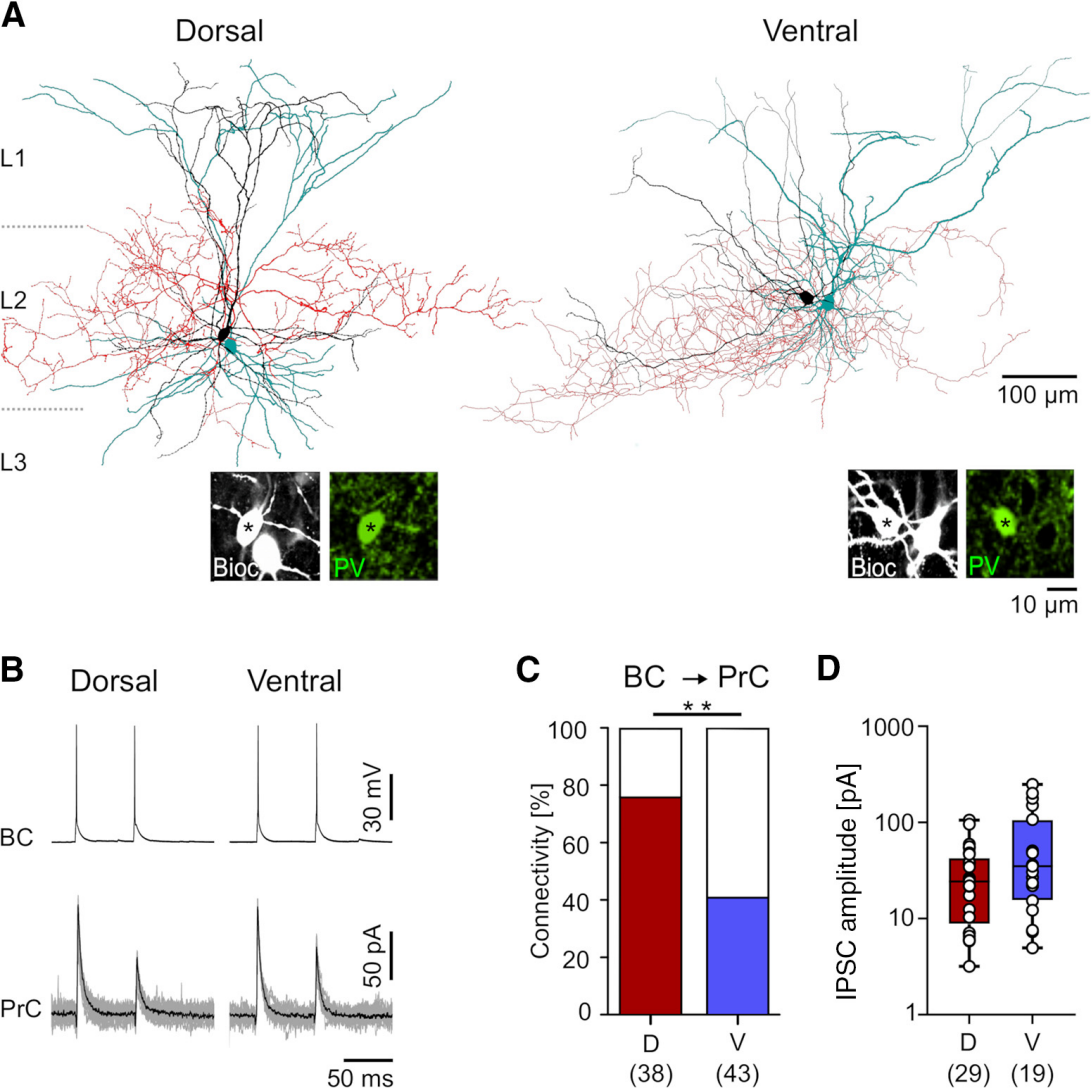
Connectivity of recorded IN-PrC pairs shows greater coupling probability in the dorsal mEC. ***A***, Morphologic reconstructions of a dorsal and a ventral synaptically-coupled PV BC-PrC pair. PV BC somata and dendrites are in black, axons in red; for PrCs only soma and dendrites are shown (in cyan); boundaries of the L1–L3 are indicated by dotted lines. Insets on the bottom illustrate the PV immunoreactivity (in green pseudocolor) in the biocytin-filled somata of the BCs (Bioc, grayscale). ***B***, Representative traces illustrate presynaptic APs evoked in PV BCs (upper traces) and short latency unitary IPSCs in concurrently recorded synaptically coupled PrCs (lower traces, averaged trace in black, individual IPSCs are superimposed in gray) in the dorsal (left) and ventral mEC (right). ***C***, Summary bar chart of the connectivity of PV BCs onto PrCs in the dorsal (D, red bars) and ventral mEC (V, blue bars). ***D***, Summary box plot of the unitary IPSC amplitudes in the dorsal (D, red bars) and ventral mEC (V, blue bars). Individual amplitude data from the pairs are superimposed as open circles on the bars. Numbers of analyzed simultaneous BC-PrC recordings are indicated in parenthesis under the bars. Statistical significance: **p < 0.01.

Given these anatomic differences, particularly in putative synapse number, we next asked whether the functional connectivity of PV BCs and PrCs was divergent between the dorsal and ventral mEC. To address this, we performed paired recordings from identified PV BCs and neighboring PrCs from both subregions (Fig. 3*A*). APs elicited in the presynaptic IN resulted in short-latency unitary IPSCs in 59% of the simultaneously recorded PrCs at −60 mV holding potential (47 connections from 81 tested pairs from 23 rats; Fig. 3*B*). Bath application of the competitive GABA_A_ agonist gabazine (10 μM) reduced these fast outward synaptic currents by 96% (peak amplitude 1.5 ± 0.1 vs 39.8 ± 22.7 pA under control conditions, 10 BC-PrC pairs, *p* = 0.026, Student’s *t* test), confirming that they were meditated by ionotropic GABA receptors. When we divided the synaptically-coupled pairs between subregions of the mEC, we found that the probability of a unitary connection from dorsal PV BCs onto local PrCs was very high at 76% (29 coupled of 38 tested pairs from 16 rats). In contrast, the connectivity of ventral mEC PV BCs onto PrCs was substantially lower at 41% among the tested pairs (18 coupled of 44 tested pairs from 14 rats; *p* = 0.001, Fisher’s exact test; Fig. 3*C*).

The amplitudes from unitary IPSCs produced by PV BCs showed a log-normal distribution, with the majority of the amplitudes occurring between 5 and 100 pA and a long tail that reached up to 250 pA. However, despite the marked differences in connectivity between dorsal and ventral mEC, the average amplitudes of unitary IPSCs with 30.6 ± 5.1 pA in the dorsal mEC and 65.4 ± 8.6 pA in the ventral mEC (Mann–Whitney *U* test; Fig. 3*D*) were not sufficiently different to reach statistical significance because of the high variability in both samples. The apparent failure rate of transmission at synapses between PV BCs and PrCs in dorsal (16.5 ± 3.8%) and ventral mEC (9.7 ± 3.3%) was comparable (*p* = 0.11, Mann–Whitney *U* test). In addition, the IPSC kinetics from both regions were also similar with respect to onset latency (dorsal: 2.2 ± 0.2 ms; ventral: 2.1 ± 0.1 ms; *p* = 0.85, Student’s *t* test), 20–80% rise time (dorsal: 0.65 ± 0.1 ms; ventral: 0.60 ± 0.1 ms; *p* = 0.47, Mann–Whitney *U* test), and decay time constant (dorsal: 5.2 ± 0.5 ms; ventral: 5.3 ± 0.6 ms; *p* = 0.33, Student’s *t* test). Finally, no difference was found in short-term plasticity in terms of paired-pulse depression (dorsal: 0.81 ± 0.03; ventral: 0.72 ± 0.04; *p* = 0.11, Student’s *t* test). Taken together, our paired recording results converge with the morphologic data demonstrating that functional synaptic connectivity is higher in the dorsal than in the ventral mEC.

**Figure 4.**
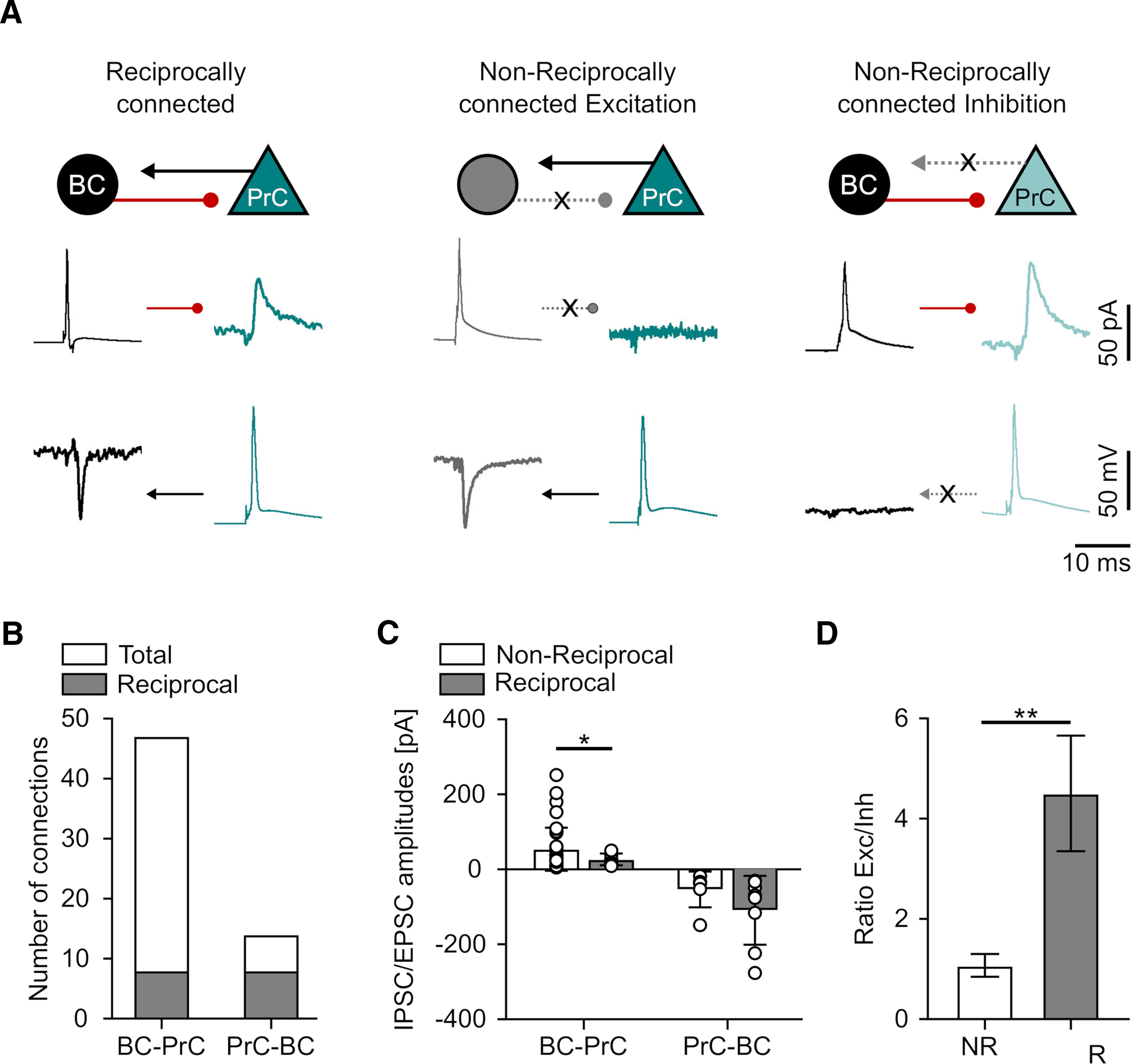
Reciprocal IN-PrC pairs in the mEC show higher excitation and lower inhibition than non-reciprocal pairs. ***A***, Schemes and representative traces illustrating the observed connectivity patterns of BC-PrC pairs: a reciprocally connected pair (left panel), a unidirectionally connected pair (middle panel) displaying only a unitary EPSC in the BC (bottom left trace, in gray) and unidirectionally connected PrC-BC pair (right panel) displaying only unitary IPSC in the PrC (top right trace in cyan). The representative presynaptic APs and the evoked unitary synaptic responses (average of 10 traces) are illustrated side-by-side in the BCs (left traces, in gray) and PrCs (right traces, in cyan). ***B***, Bar chart of the total number of connected PV BC and PrC pairs (reciprocal connections, in gray). ***C***, Summary bar chart of the unitary IPSC and EPSC amplitudes for reciprocal and non-reciprocal connections. Individual peak amplitude data from the pairs are superimposed as open circles on the bars. ***D***, Ratio of excitation and inhibition in non-reciprocal (NR, white bar) versus reciprocal pairs (R, gray bar). Statistical significance: *p < 0.05, **p < 0.01.

In 8 of the simultaneously recorded pairs (5 in the dorsal and 3 in the ventral mEC), we observed that connectivity between BCs and PrCs was reciprocal (Fig. 4*A*,*B*). These reciprocally connected pairs had smaller mean unitary IPSC (24.6 ± 15.6 pA) than the non-reciprocally connected BC-PrC pairs (51.7 ± 57.5 pA, 39 pairs, *p* = 0.015, Student’s *t* test with Welch’s correction; Fig. 4*A*,*C*). We observed no statistical difference in EPSC amplitude of reciprocally connected BC-PrC pairs (−110.8 ± 91.9 pA) compared with non-reciprocal BC-PrC pairs (−55.6 ± 47.6 pA, 6 pairs, *p* = 0.21, Mann–Whitney *U*). Consequently, the ratio of excitation *versus* inhibition for reciprocal connections with 4.5 ± 4.6 was considerably higher than the ratio of EPSC and IPSC amplitudes for non-reciprocally connected neurons (1.1 ± 1.4, *p* = 0.01; Fig. 4*D*). These results suggest that mEC PV BC microcircuits might be wired for competitive interactions whereby selected PrCs involved in reciprocally coupling with BCs are able to effectively recruit perisomatic inhibition to suppress neighboring PrCs.

### Connectivity onto identified PCs and SCs

**Figure 5.**
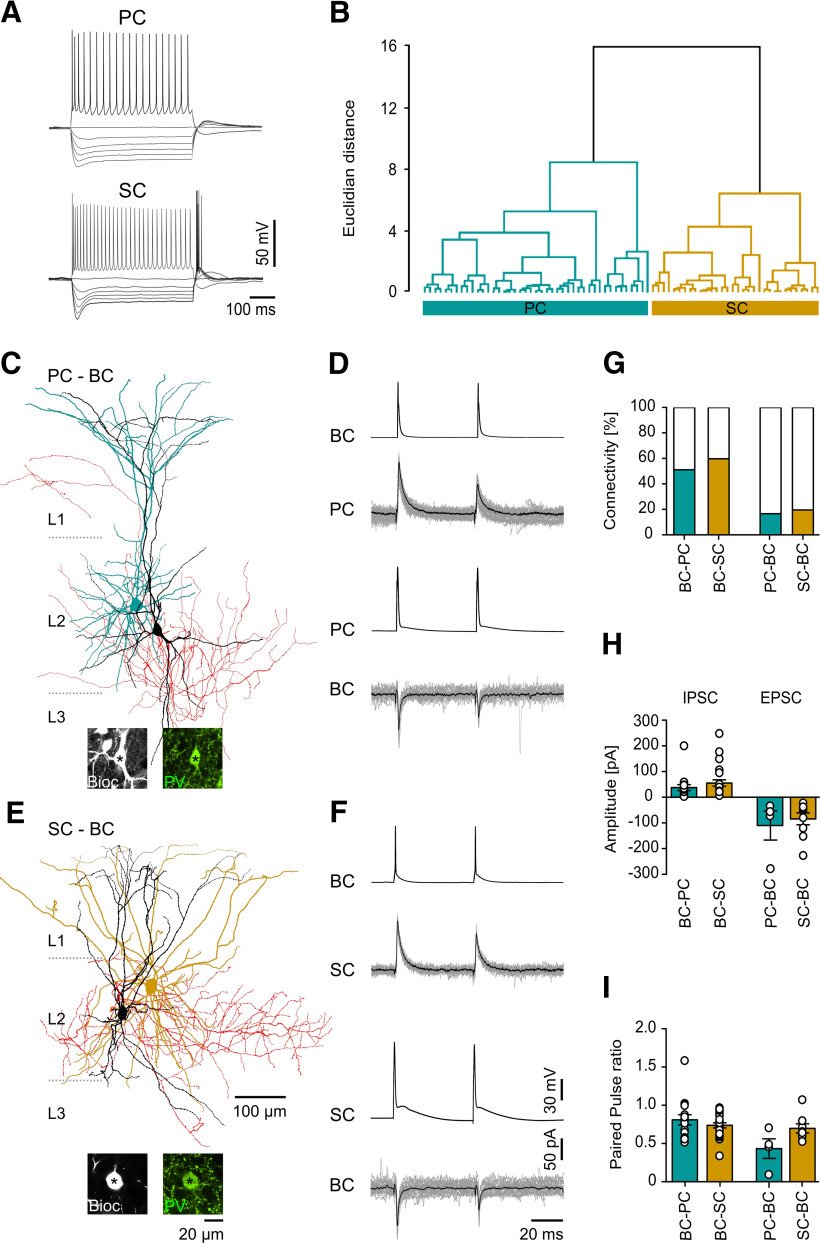
Properties of synaptic coupling of SCs and PCs to PV BCs. ***A***, Voltage responses of a PC (top) and a SC (bottom) to hyperpolarizing (−500 to −100 pA, 500 ms in duration) and a suprathreshold depolarizing current pulse (500 pA). ***B***, Dendrogram illustrates the separation of two subpopulations of PrCs corresponding to PCs (in cyan) and SCs (in ochre) by cluster analysis. ***C***, ***E***, Morphologic reconstructions of a synaptically-coupled PV BC-PC (***C***) and a PV BC-SC pair (***E***). PV BC somata and dendrites are in black, axons in red; for the PrCs only soma and dendrites are shown (PC in cyan; SC in ochre); boundaries of L1–L3 are indicated by dotted lines. Insets on the bottom illustrate the PV immunoreactivity (in green pseudocolor) in the biocytin-filled IN somata (Bioc, grayscale). ***D***, Electrophysiological data from a reciprocally connected BC-PC pair. Representative trace illustrates presynaptic APs evoked in a PV BC (top trace) and the short latency unitary IPSCs in the concurrently recorded PC (upper middle trace, averaged response in black, individual IPSCs are superimposed in gray). Similarly, presynaptic APs evoked in the PC (lower middle trace) were followed by short latency unitary EPSCs in the concurrently recorded IN (bottom trace). ***F***, Electrophysiological data from a reciprocally connected BC-SC pair as illustrated in ***D***. ***G***, Summary bar chart of the connection probabilities between PV BCs and PCs and SCs in the mEC. ***H***, ***I***, Bar charts of the amplitudes (***H***) and paired pulse ratios (***I***) of unitary IPSCs and EPSCs between PV BCs and synaptically coupled PCs and SCs. Individual values from the pairs are superimposed as open circles on the bars.

It has previously been shown that different PrC subtypes, PCs and SCs, receive differential input from PV BCs in the mouse (Fuchs et al., 2016). Therefore, we next asked whether we observed similar differences in target cell specificity in our sample of PV BC-PrC pairs in juvenile rats. Simultaneously recorded PrCs were classified, grouping them based on their morphology and physiological properties (Fig. 5*A*; Table 3) by applying a cluster analysis as previously described (Fuchs et al., 2016; Fig. 5*B*). In good agreement with clearly identifiable examples of PCs and SCs among our reconstructed PrCs (Fig. 5*C*,*E*), we observed two well-defined clusters emerging in this analysis, which corresponded to PCs (35 cells) and SCs (40 cells; Fig. 5*B*). In 7 PrCs, there was insufficient data to perform the cluster analysis and these cells were excluded from further analysis. We did not observe clustering corresponding to intermediate cell types as previously reported in mice (Fuchs et al., 2016).

**Table 3:** Passive and active physiological properties of stellate and pyramidal PrCs

	SCs	PCs	*p* value
Passive properties			
Resting membrane potential (mV)	−59.4 ± 0.8 (22)	−62.0 ± 1.2 (23)	0.06
Input resistance (MΩ)	94.3 ± 11.6 (22)	162.0 ± 20.0 (23)	<0.01
Membrane time constant (ms)	13.3 ± 0.8 (21)	23.7 ± 2.4 (20)	<0.001
Membrane capacitance (pF)	185.7 ± 23.4 (20)	209.5 ± 35.5 (21)	0.51
Active properties			
Rheobase (pA)	90.9 ± 10.2 (22)	89.1 ± 11.7 (23)	0.91
Voltage threshold (mV)	−39.8 ± 0.8 (22)	−41.7 ± 1.0 (23)	0.28
AP amplitude (mV)^*^	78.1 ± 1.6 (22)	83.8 ± 1.9 (23)	<0.05
AP maximum rise rate (mV/ms)	346.1 ± 19.4 (22)	433.3 ± 31.8 (23)	<0.05
AP maximum decay rate (mV/ms)	111.1 ± 19.1 (22)	81.8 ± 3.8 (23)	0.15
Half-height duration (ms)	0.92 ± 0.03 (22)	1.11 ± 0.05 (23)	<0.05
Fast AHP amplitude (mV)^*^	−10.7 ± 0.4 (22)	−10.4 ± 0.6 (23)	0.65
Discharge frequency at 250 pA (Hz)	34.0 ± 2.9 (22)	39.7 ± 2.6 (23)	0.15
Voltage sag (mV)^**^	−9.2 ± 0.8 (38)	−6.3 ± 0.8 (32)	<0.05
ISI ratio^**^	0.36 ± 0.05 (38)	0.61 ± 0.05 (32)	<0.01
dAP (mV)^**^	2.4 ± 0.3 (38)	1.6 ± 0.3 (32)	0.13
Latency to first AP at 205 pA (ms)^**^	19.6 ± 3.3 (38)	46.8 ± 8.4 (32)	<0.01

Results shown as mean ± SEM, number of analyzed neurons shown in parenthesis. Parameters used for the principal component analysis are highlighted with a double asterisk.

* Measured from threshold.

** Parameter used for PCA. ISI ratio was calculated from the first and the second interval.

Analysis of the synaptic connectivity between PV BCs and PrCs on the basis of this classification demonstrated that the connection probability was independent of whether the postsynaptic target was a PC (Fig. 5*C*,*D*) or a SC (Fig. 5*E*,*F*). The average connection probability onto PCs was 55% (18 coupled of 35 tested pairs from 20 rats) and onto SCs it was comparable at 60% (24 coupled of 40 tested pairs from 17 rats; *p* = 0.66, Fisher’s exact test; Fig. 5*G*). In contrast, the connection probability from PrCs onto PV BCs was substantially lower at 17% for PCs (six coupled of 35 tested pairs) and similarly low at 20% for SCs (eight coupled of 40 tested pairs, *p* = 0.78, Fisher’s exact test; Fig. 5*G*).

Finally, we examined whether the connectivity and synaptic properties of PV BCs onto PrC subtypes was different between dorsal and ventral mEC. From dorsal PV BCs we found connection probabilities of 77% onto PCs (10 coupled of 13 tested pairs from eight rats) and 78% onto SCs (14 coupled of 18 tested pairs from 11 rats), which was not different between the two target cell types (*p* = 1.0, Fisher’s exact test). The connection probability of ventral PV BCs was 36% onto PCs (eight coupled of 22 tested pairs from 12 rats) and 46% (10 coupled of 22 tested pairs from 11 rats) onto SCs, consistent with the overall lower probability of connectivity in this region (see above), but with no significant difference between the two PrC subtypes (*p* = 0.54, Fisher’s exact test).

We observed no overt differences in any underlying property of the unitary synaptic responses recorded in classified PrC subtypes, as the amplitude of IPSCs recorded in SCs was 55.2 ± 12.8 pA and in PCs was 37.7 ± 11.8 pA (*p* = 0.35, Student’s *t* test). Similarly, the amplitude of EPSCs in PV BCs was similar whether they resulted from inputs from SCs (−83.4 ± 22.8 pA) or from PCs (−108.7 ± 56.8 pA; *p* = 0.83, Mann–Whitney *U* test; Fig. 5*H*). Finally, there was no observed statistical difference in the IPSC paired-pulse ratio produced by INs (IN-SC: 0.74 ± 0.03, IN-PC: 0.81 ± 0.07, *p* = 0.59) or EPSCs (SC-IN: 0.69 ± 0.06, PC-IN: 0.44 ± 0.13, *p* = 0.09, Mann–Whitney *U* test; Fig. 5*I*).

Together, these data show that the reciprocal connectivity between PV BCs and PrCs is independent of PrC type in the juvenile rat. However, the observed differences in the connection probability of PV BCs onto PrCs along the dorsoventral axis may enable these INs to produce more robust lateral inhibition in dorsal mEC microcircuits.

## Discussion

In this study we provide a comprehensive characterization of L2/3 PV BCs along the dorsoventral axis of the mEC, with respect to their intrinsic physiology, morphology, and connectivity. While we observed minimal differences in PV BC intrinsic physiology or dendritic and axonal morphology along this axis, PV BCs were more synaptically coupled to the local microcircuit in the dorsal compared with ventral mEC, plausibly explaining divergent lateral inhibition observed in previous studies (Beed et al., 2013).

### Morphology and intrinsic physiology of PV BCs does not account for divergent mEC inhibition

INs comprise ∼20% of all neurons in the mEC, of which fast-spiking PV BCs represent ∼50% and as such exert powerful control over local network activity in L2/3 (Jones and Bühl, 1993; Beed et al., 2013; Pastoll et al., 2013). In the neocortex, fast-spiking PV BCs have been described physiologically (Martin et al., 1983; Gupta et al., 2000; Wang et al., 2002), morphologically (Kisvárday et al., 1985; Thomson et al., 1996) and with respect to their connectivity and network functions (White, 1989; Dantzker and Callaway, 2000; Cunningham et al., 2004). By contrast, in the mEC, PV BCs have not been thoroughly studied. Here, we describe the intrinsic physiological and morphologic characteristics of INs in L2/3 in both the dorsal and ventral mEC, which were all putatively identified as BCs based on their immunoreactivity, axonal distribution and formation of boutons around neighboring neuronal somata. Despite heterogeneity in their morphology, there were no major systematic differences in their properties along the mEC dorsoventral axis that would account for divergent lateral inhibition (Beed et al., 2013). In cortical areas, at least five subpopulations of PV INs have been described: large BCs, narrow BCs, nest BCs, clutch cells, and axo-axonic cells which each possess different physiological and morphologic properties (Somogyi et al., 1983; Kisvárday et al., 1985; Jones and Bühl, 1993; Kawaguchi and Kubota, 1997; Dantzker and Callaway, 2000; Pastoll et al., 2013; Buetfering et al., 2014). We excluded putative axo-axonic cells, and did not attempt to characterize PV BCs based on these subgroups, which may contribute to some morphologic heterogeneity in our data. Despite this, the absence of distinct morphologic divergence along the dorsoventral mEC suggests that the increased lateral inhibition and PV immunolabeling observed in the dorsal mEC (Beed et al., 2013) must arise from an alternative mechanism, such as the divergent axon bouton density and connectivity that we describe here.

### Differential connectivity of mEC PV BCs along the dorsoventral axis

Our data reveal that PV BCs in the supragranular layers of the mEC innervate local PrCs to a greater extent in the dorsal, as compared with ventral mEC. This higher inhibitory connectivity formed by PV BCs plausibly leads to more robust inhibition in PrCs at dorsal levels. Indeed, Beed et al. (2013) showed that while the maximum distance of inhibitory input points is broadly similar (∼500 μm), the relative strength over the entire local region is ∼50% higher in dorsal than ventral SCs. This finding fits well to our data, showing that, the axonal arbors of PV INs spread up to 300–500 μm from the soma in both subregions; however, the connection probability is ∼50% higher in dorsal environs. This higher connection probability correlates with a higher density of boutons along axon collaterals. A complementary factor which might further underlie higher connection probabilities is that dorsal PrC somata have been shown to be larger, and thus might be contacted by more PV terminals than ventral somata (Berggaard et al., 2018). The higher density of varicosities along BC axons is in good agreement with previous findings that the volume density of PV axon terminals is decreasing along the dorsoventral axis, whereas the density of PV somata shows only a moderate decline (Beed et al., 2013), also suggesting a higher number of presynaptic terminals formed by dorsal *versus* ventral PV BCs. However, other studies found a steeper decline in the number of PV somata in ventral than in dorsal sections of mEC in the rat (Kobro‐Flatmoen and Witter, 2019) and in mice (Fujimaru and Kosaka, 1996). Thus, a lower divergence of the synaptic output of PV BCs, a lower convergence onto the target PrCs and a lower number of the INs may lead to weaker inhibition in the ventral compared with the dorsal mEC subregion.

It has been previously shown that PrCs segregate into four defined subtypes with differential connectivity to local INs in the mouse (Fuchs et al., 2016). In our cluster analysis of mEC PrCs performed in rats using the same parameters, we only observed two distinct clusters corresponding to PC and SCs. Furthermore, connectivity analysis of the identified two PrC clusters did not reveal differential connectivity onto PCs versus SCs from PV BCs in the rat. A plausible explanation for this divergence beyond a simple species difference could be a difference in the age of the animals: juvenile in this study and more mature mice in Fuchs et al. (2016), which will require further study.

With respect to the dorsoventral differences among PrCs, it has been recently shown that the intrinsic physiology of SCs favors a greater voltage response of these neurons to depolarizing currents in the ventral mEC, with minimal divergence in the properties of PCs (Pastoll et al., 2020). How the interplay between higher AP discharge probabilities from ventral SCs affects the recruitment of PV BCs and the generation of feedback inhibition during grid field firing remains yet to be analyzed.

### A role for PV BCs in the generation of divergent spatial tuning in dorsal and ventral mEC?

A key role which has been proposed for the superficial layers of the mEC is to encode spatial information, with most neurons showing some degree of spatial tuning in their activity (Couey et al., 2013; Tennant et al., 2018). Most prominent examples of PrCs with such spatially-modulated discharge observed in this region are grid cells (Hafting et al., 2005) and head-direction cells (Sargolini et al., 2006; Tang et al., 2014). Feedback inhibitory loops, involving fast-spiking PV BCs in interaction with PrCs, has been identified as a potential mechanism for the generation of spatially controlled PrC grid activity in *in vivo* electrophysiological and optogenetic experiments (Fyhn et al., 2007; Couey et al., 2013; Pastoll et al., 2013; Shipston-Sharman et al., 2016; Miao et al., 2017). Network simulations using attractor-based models further underlined the importance of feedback inhibition for the generation of grid cell activity (Fyhn et al., 2007; Couey et al., 2013; Pastoll et al., 2013; Shipston-Sharman et al., 2016; Miao et al., 2017) despite the fact that PV BCs themselves appear to have only minimal spatial tuning (Buetfering et al., 2014). One major divergence in the phenomenology of coherent grid activity, as a proxy for spatial information processing, is the presence of a gradient of grid sizes, with smaller spatial fields observed in the dorsal mEC and larger grids in the ventral mEC (Naumann et al., 2016). Our data provides evidence for the involvement of PV BC-mediated inhibition in such a divergent grid cell firing, as high connectivity will provide stronger inhibition and tighter spatial tuning as observed in the dorsal mEC. Conversely, low connectivity would allow PrCs to discharge over a wider spatial field, or in response to reduced depolarizing currents (Pastoll et al., 2020), as observed in the ventral mEC. Convergently, gamma frequency oscillations (20–200 Hz) have been shown to be stronger in the dorsal mEC (Beed et al., 2013). Given the defined role of PV BCs in the generation of emergent gamma oscillations (Bartos et al., 2002, 2007; Tukker et al., 2007), the stronger inhibition provided by their higher connectivity in the dorsal mEC may also provide a cellular substrate for this difference in population activity.

In summary, we provide the first thorough description of the morphology, physiology and connectivity of PV BCs in the rat mEC, with respect to the dorsoventral axis. We show that while PV BC morphology and intrinsic physiology is similar along this axis, the connectivity of PV BCs is highest in dorsal and lowest in the ventral mEC, plausibly contributing to differences in spatial coding within these two regions.

## Synthesis

Reviewing Editor: Katalin Toth, University of Ottawa Decisions are customarily a result of the Reviewing Editor and the peer reviewers coming together and discussing their recommendations until a consensus is reached. When revisions are invited, a fact-based synthesis statement explaining their decision and outlining what is needed to prepare a revision will be listed below. The following reviewer(s) agreed to reveal their identity: Desdemona Fricker.

The manuscript describes single cell properties and connectivity patterns of PV interneurons in the dorsal and ventral mEC. Both reviewers thought that the study is carefully designed and executed, the experiments are technically solid and the findings will provide valuable;e information about the network connectivity pattern in this region. Both reviewers provided constructive comments that should help the authors to further improve their manuscript through increased precision and few points of clarifications.

Rev.#1.

In this study the Authors have investigated the connectivity of PV INs and PrC in the mEC along the dorso-ventral axis. One of the main findings of the study is that the morphological and single cell features of PV INs are comparable in the dorsal and ventral mEC. The other main finding is that the probability of finding a connection between PV INs and PrC is significantly higher in the dorsal mEC than in the ventral mEC. My comments are the followings.

1) The Authors claimed that PV INs what they included in the study had morphological features of basket cells. Therefore, it is misleading to name these interneurons as PV INs. If one knows the morphology of interneurons, then it is more appropriate to name the recorded interneurons as PV basket cells. In the cortex, chandelier cells can be distinguished clearly from basket cells based on the morphology of axonal arbor. I would highly encourage the Authors i) restrict their analysis to basket cells and ii) separate chandelier cells from basket cells and report their number recorded in this study. It is not needed to distinguish all distinct types of basket cells have been described in earlier studies, pooling data into a single basket cell category is a perfect choice.

2) The Authors reported that the reobase of PV INs is around 200 pA (Table 2). Yet, both examples shown in Fig. 1D and E have no action potentials at current steps of 500 pA. Are these examples exceptions or the calculation of the reobase values should be double-checked?

3) Could the Authors report the occurrence of reciprocally connected pairs? If these reciprocally connected pairs had a substantial number, could the Authors plot the peak amplitude of EPSCs vs peak amplitude of IPSCs recorded in the reciprocally connected pairs? The rationale beyond this investigation is that those reciprocally connected pairs that belong to a functional neural ensemble tend to be reciprocally connected with larger unitary events. If the present data set is large enough, then it might be of interest to see whether such a relation is present in the microcircuits of the mEC.

4) The Authors report that the probability of connections between PV INs and PrCs is higher in the dorsal than in the ventral mEC, in spite of the fact that PV INs in both regions have axonal arbors with a similar extent. This statement should be strengthened by showing that the density of the neurons is actually the same in the area of the axonal arbor of PV INs in both regions. It could occur, however, that the number of neurons is significantly higher in a given volume in the ventral mEC in comparison to that present in the dorsal mEC, giving rise to lower chance to find a connected pair in the ventral mEC, if the divergence of PV INs in both regions is comparable.

Rev. #2.

The present manuscript focuses on PV interneurons in the superficial layers 2/3 of the medial Entorhinal Cortex (mEC). The authors provide a characterization of their intrinsic electrophysiological and morphological properties. They determine connection probabilities between PV cells and two types of principal neurons, the stellate cells and pyramidal cells, using paired recordings. All experiments were performed in either the dorsal or ventral portion of the mEC. While most examined parameters were found to be similar between dorsal and ventral sections, the connection probability from PV cells onto local principal neurons was higher dorsally, and there was also a higher density of presynaptic boutons on the axon of PV neurons in dorsal mEC. This dichotomy fits with previous studies showing a dorso-ventral inhibitory gradient in the mEC.

The study is based on slice experiments combining patch clamp recordings and morphological analysis. Some methods are not well documented, this needs to be improved. Reconstructions are of high quality. While the results and conclusions are not entirely novel, the comparison of dorsal vs ventral sections with a carefully documented set of experiments constitutes a unique contribution to the literature on mEC inhibitory microcircuits. Inhibition is thought to be important for the generation of grid-cell activity in mEC, and the gradient in inhibition mirrors a gradient in grid field size. In view of the central position of this brain area in the spatial navigation system, the present work will be of interest to a broad neuroscience audience.

Major points:

1/ p9, line 220 ff. Data on the excitatory synaptic input onto PV cells is missing. The text says that amplitudes and frequencies of spontaneous EPSCs were measured. Table 1 does however not include these data. This should be corrected, or, even better, instead of including the numbers in the table, a figure with the amplitude distributions and frequency distributions would be good. Please also show sample traces for each condition.

2/ p11, line 289. Data on amplitudes from unitary IPSCs should be added to Figure 3. A graph with amplitude distributions would be good to illustrate the log-normal distribution.

3/ Figure 4H. Amplitudes of IPSCs and EPSCs should surely be measured in pA (y axis).

4/ Figure 4D. What was the holding potential in the voltage clamp traces in paired recordings? I am a bit doubtful whether the method section is complet: there is only 1 internal solution given, that is based on K gluconate and low Cl. On page 5 line 126, the paper makes reference to anther paper that is blanked. If there is another internal solution that has been used in this ms, the authors should give the composition of all the internal solutions, and indicate the holding potentials in the recordings. A reference to a previous paper is not enough.

5/ The methods for cluster analysis (Fig 4B) separating SC and Pyr cells must be documented in the methods section. The parameters that were used for clustering must be listed. Please also add a table with the electrophysiological active and passive properties of the recorded excitatory cells.

6/ The authors seem to have a data set of at least 43 reconstructed PV cells. How many principal neurons (PC or SC) were reconstructed? Can the authors make the reconstructions available and include the full data set of reconstructed neurons as a supplementary figure?

7/ DAPI seems to be in green not in white in Figure 1.

8/ Figure 4C. Is the scale bar the same as in Fig 4E? The limit between layer 1 and layer 2 (dotted line) seems surprisingly low with respect to the neurons’ arborizations/the apical tuft. Can you please double check if this is correct.
